# WD Repeat Protein WDR48 in Complex with Deubiquitinase USP12 Suppresses Akt-dependent Cell Survival Signaling by Stabilizing PH Domain Leucine-rich Repeat Protein Phosphatase 1 (PHLPP1)*

**DOI:** 10.1074/jbc.M113.503383

**Published:** 2013-10-21

**Authors:** Narmadha Reddy Gangula, Subbareddy Maddika

**Affiliations:** From the Laboratory of Cell Death and Cell Survival, Centre for DNA Fingerprinting and Diagnostics (CDFD), Nampally, Hyderabad 500001, India

**Keywords:** Akt, Apoptosis, Protein Stability, Tumor Suppressor Gene, Ubiquitin, PHLPP, USP12, WDR48

## Abstract

PHLPP1 (PH domain leucine-rich repeat protein phosphatase 1) is a protein-serine/threonine phosphatase and a negative regulator of the PI3-kinase/Akt pathway. Although its function as a suppressor of tumor cell growth has been established, the mechanism of its regulation is not completely understood. In this study, by utilizing the tandem affinity purification approach we have identified WDR48 and USP12 as novel PHLPP1-associated proteins. The WDR48·USP12 complex deubiquitinates PHLPP1 and thereby enhances its protein stability. Similar to PHLPP1 function, WDR48 and USP12 negatively regulate Akt activation and thus promote cellular apoptosis. Functionally, we show that WDR48 and USP12 suppress proliferation of tumor cells. Importantly, we found a WDR48 somatic mutation (L580F) that is defective in stabilizing PHLPP1 in colorectal cancers, supporting a WDR48 role in tumor suppression. Together, our results reveal WDR48 and USP12 as novel PHLPP1 regulators and potential suppressors of tumor cell survival.

## Teaser

**Background:** PHLPP1 is a tumor suppressor phosphatase.

**Results:** WDR48 in complex with USP12 controls cell survival signaling by deubiquitinating PHLPP1.

**Conclusion:** WDR48 acts as a potential tumor suppressor by positively regulating PHLPP1 stability.

**Significance:** WDR48 was identified as novel PHLPP1 regulator, which is critical in understanding its role as tumor suppressor in human cancers.

## Introduction

The phophatidylinositol 3-kinase/Akt pathway is known to be hyperactivated in many human cancers (1). Many molecular players in this pathway are reported to act as either tumor suppressors or oncogenes. PHLPP1^3^ is known to negatively regulate Akt by direct dephosphorylation and play an important role in cell survival, proliferation, migration, and cell death (2, 3). The loss of PHLPP1 is reported in various cancers such as colon (4), breast (5), ovarian (6), Wilms tumors (5), prostate (7), and hepatocellular carcinomas (8). Overexpression of PHLPP1 in glioblastoma and colon cancer cells inhibits tumorigenesis in xenografted nude mice (2, 9), whereas decreased PHLPP1 expression correlates with increased metastatic potential in breast cancer cells (5). In addition, PHLPP1 is localized on chromosome 16q22.3, a region that encounters frequent loss of heterozygosity in many primary and malignant breast tumors (10). Also, PHLPP1 levels are markedly reduced in several cancer cell lines that have elevated Akt phosphorylation, and the reintroduction of PHLPP1 reduces cell growth by inactivating the Akt pathway (8). Apart from Akt dephosphorylation, PHLPP1 has additional substrates such as MST1, PKC, and S6K1, which are all critical for PHLPP1 tumor suppressor function.

The crucial function of PHLPP1 in controlling cell survival and its involvement in human malignancies suggest that the enzyme needs to be tightly regulated *in vivo*. Regulation of PHLPP1 protein stability via ubiquitination was recently identified as one of the mechanisms to control PHLPP1 function in cells. β-TrCP-containing Skp·Cullin 1·F-box protein (SCF) complex (SCF^β-TrCP^) was identified as an E3 ubiquitin ligase that ubiquitinates and degrades PHLPP1 in a phosphorylation-dependent manner (11). On contrary, subsequent studies have identified USP1 (12) and USP46 (13) as deubiquitinases that can hydrolyze ubiquitin chains on PHLPP1 and thus stabilize the protein. Given that PHLPP1 acts as a tumor suppressor in various cancers derived from different cellular origins, these regulators may not fully elucidate the molecular players controlling PHLPP1 function. Thus, in this study we attempted to identify additional regulators of PHLPP1 by using tandem affinity purification followed by proteomic analysis. We revealed WDR48 as an additional regulator of PHLPP1 and a potential tumor suppressor. Our biochemical studies have shown that WDR48 recruits USP12 deubiquitinase to catalyze the removal of ubiquitin chains on PHLPP1 and thus positively regulates the PHLPP1 stability.

## EXPERIMENTAL PROCEDURES

### 

#### 

##### Plasmids

Full-length PHLPP1, USP1, USP7, USP12, USP46, WDR48, WDR5, domain deletion mutants of PHLPP1, deletion mutants of WDR48, and PHLPP1 ΔWIR mutant (interaction-deficient) were PCR-amplified and cloned into S-protein/FLAG/streptavidin-binding protein (SFB)-triple-tagged destination vector using the Gateway cloning system (Invitrogen). Full-length WDR48 and USP12 were cloned to a Myc-tagged destination vector. HA-PHLPP1 and GST-tagged PHLPP1 vectors were generated by transferring their coding sequences into destination vectors using the Gateway system. USP12 catalytically inactive mutant (C48S) and WDR48 mutants were generated by PCR-based site-directed mutagenesis.

##### Antibodies

Anti-PHLPP1 (Bethyl Laboratories), anti-USP12, anti-GST, anti-Myc, clone 9E10 (all from Santa Cruz Biotechnologies), anti-pAkt (Ser-473), anti-Akt, anti-phospho-p70 S6K (Thr-389), anti-p70 S6K, anti-phospho-S6 (Ser-235/Ser-236), anti-S6 (Cell Signaling Technologies), anti-WDR48, anti-FLAG, anti-actin, anti-HA (all from Sigma), and anti-ubiquitin (Millipore) antibodies were used in this study.

##### Tandem Affinity Purification

PHLPP1-associated proteins were isolated by using tandem affinity purification as described before (14). Briefly, 293T cells were transfected with SFB-triple-tagged PHLPP1, and 48 h later cells were lysed with NETN buffer (20 mm Tris-HCl, pH 8.0, 100 mm NaCl, 1 mm EDTA, 0.5% Nonidet P-40) containing 50 mm β-glycerophosphate, 10 mm NaF, 1 μg/ml of each pepstatin A, and aprotinin on ice for 30 min. After removal of cell debris by centrifugation, crude cell lysates were incubated with streptavidin-Sepharose beads (Amersham Biosciences) for 1 h at 4 °C. The bound proteins were washed three times with NETN and then eluted twice with 2 mg/ml biotin (Sigma) for 60 min at 4 °C. The eluates were incubated with S-protein-agarose beads (Novagen) for 1 h at 4 °C and then washed three times with NETN. The proteins bound to S-protein-agarose beads were eluted by boiling in SDS-loading dye and then resolved by SDS-PAGE. The identities of eluted proteins were revealed by mass spectrometry analysis performed by the Taplin Biological Mass Spectrometry Facility at Harvard.

##### Cell Transfections, Immunoprecipitation, and Immunoblotting

HEK 293T, HeLa, and HCT116 cells were maintained in RPMI 1640 medium supplemented with 10% FBS. Cells were transfected with various plasmids using polyethyleneimine transfection reagent. For immunoprecipitation assays, cells were lysed with NETN buffer. The whole cell lysates obtained by centrifugation were incubated with 2 μg of specified antibody bound to either protein A or protein G-Sepharose beads (Amersham Biosciences) for 1 h at 4 °C. The immunocomplexes were then washed with NETN buffer four times and applied to SDS-PAGE. Immunoblotting was performed following standard protocols.

##### GST Pulldown Assays

Bacterially expressed GST-PHLPP1 or control GST bound to glutathione-Sepharose beads (Amersham Biosciences) were incubated with 293T cell lysates for 1 h at 4 °C. The washed complexes were eluted by boiling in SDS sample buffer and separated by SDS-PAGE, and the interactions were analyzed by Western blotting.

##### In Vivo Ubiquitination Assay

Cells were transfected with various combinations of plasmids as indicated in Figs. 3 and 6, and 24 h after transfection cells were treated with MG132 (10 μm) for 6 h. The whole cell extracts prepared by NETN lysis were subjected to immunoprecipitation of the substrate protein. The analysis of ubiquitination was performed by immunoblotting with ubiquitin antibody.

##### RNA Interference

The vector containing WDR48 shRNA (5′-AATAACATAGGAAACCTGCAC-3′) and USP12 shRNA (5′-AAACAGACGAAGTTCTAAAGG-3′) was transfected, and 48 h after transfection the cells were collected, and the efficiency of knockdown was checked by immunoblotting with specific antibodies.

##### Apoptosis Assay

HCT116 cells were transfected with various indicated vectors. Twenty-four hours after transfection the cells were washed with PBS and then treated with propidium iodide-hypotonic lysis buffer (0.1% sodium citrate, 0.1% Triton X-100, 100 μg/ml RNase, 50 μg/ml propidium iodide). After 30 min of incubation, the samples were analyzed by flow cytometry, and the percentage of apoptotic cells was calculated based on the sub-G_1_ peak.

##### Cell Proliferation Assay

HCT116 cells were transfected as required and then seeded into 100-mm or 60-mm dishes. The rate of cell proliferation was analyzed by counting the viable cells after staining them with trypan blue at the indicated times.

## RESULTS

### 

#### 

##### WDR48 and USP12 Are Novel PHLPP1-associated Proteins

**FIGURE 1.**
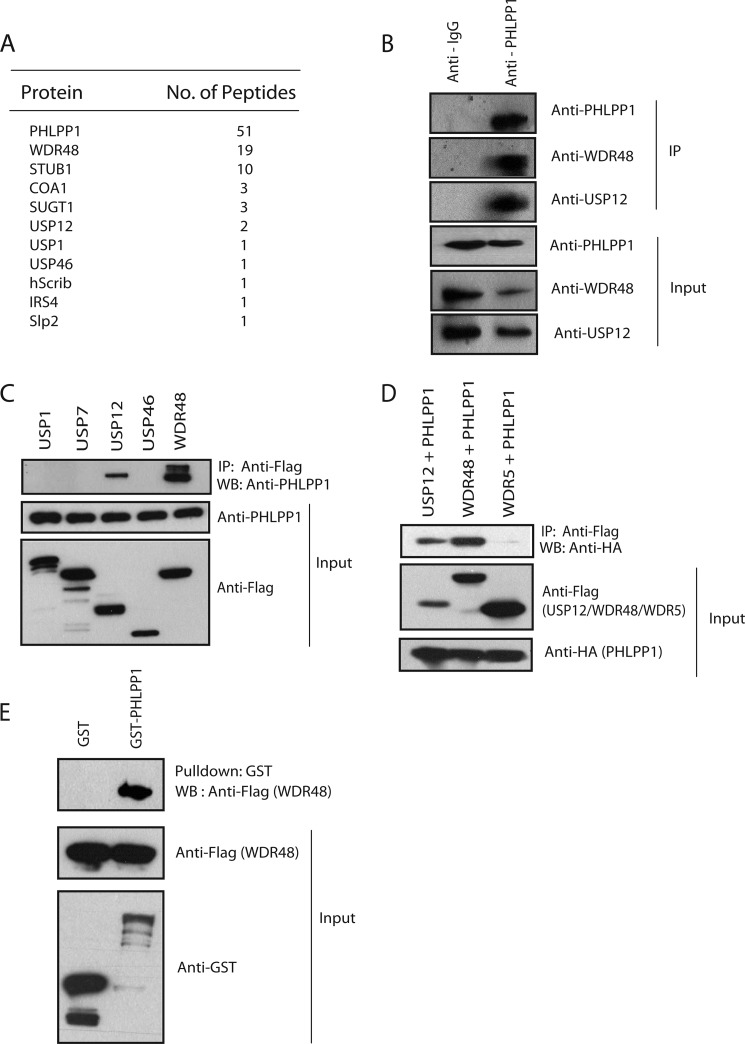
**WDR48 and USP12 are PHLPP1-associated proteins.** *A*, PHLPP1 was used as the bait for tandem affinity purification followed by mass spectrometry analysis. The proteins identified in the PHLPP1-containing complexes are listed together with the number of peptides for each protein identified by mass spectrometry. *B*, immunoprecipitation (*IP*) with either control IgG or PHLPP1 antibody was performed, and the association of endogenous PHLPP1 with WDR48 and USP12 was evaluated by immunoblotting with WDR48 and USP12 antibodies. *C*, 293T cells were transfected with the indicated FLAG-tagged plasmids, and the interaction of endogenous PHLPP1 with WDR48 and USP12 was evaluated by immunoprecipitation with FLAG antibody followed by immunoblotting (*WB*) with PHLPP1 antibody. *D*, 293T cells were co-transfected with HA-tagged PHLPP1 along with FLAG-tagged USP12, WDR48, and WDR5. Immunoprecipitation using anti-FLAG antibody was performed, and the interaction of PHLPP1 with WDR48 and USP12 was evaluated by immunoblotting with the HA tag-specific antibodies. *E*, GST pulldown assay was performed using immobilized control GST or GST-PHLPP1 fusion proteins on agarose beads followed by incubation with extracts prepared from 293T cells expressing SFB-WDR48. The *in vitro* interaction of WDR48 with PHLPP1 was assessed by immunoblotting with anti-FLAG antibody.

In an attempt to identify molecular players involved in regulation of PHLPP1 during its tumor suppressor function, we transfected 293T cells with a triple epitope (S-protein, FLAG, and streptavidin-binding peptide)-tagged version of PHLPP1 (SFB-PHLPP1). Tandem affinity purification from the lysates of these cells using streptavidin-agarose beads and S-protein-agarose beads followed by mass spectrometry analysis was performed. We identified WDR48 as one of the major associated proteins in the PHLPP1 complex (Fig. 1*A*) in addition to several previously identified PHLPP1-associated proteins such as hScrib (15) and SUGT1 (16). WDR48 (also known as p80 or UAF1) is a WD40 repeat-containing protein that was originally identified as a regulator of Fanconi anemia pathway in association with deubiquitinating enzyme USP1 (17). Subsequent studies have shown that WDR48 is an integral component and an activator of other deubiquitinases such as USP12 and USP46 (18). Although proteins such as FANCD2, PCNA, notch receptor, and histones H2A and H2B were reported to be deubiquitinated by the WDR48·DUB complex (17, 19–21), the cellular functions of WDR48 and its role in tumor suppression were not completely known.

**FIGURE 2.**
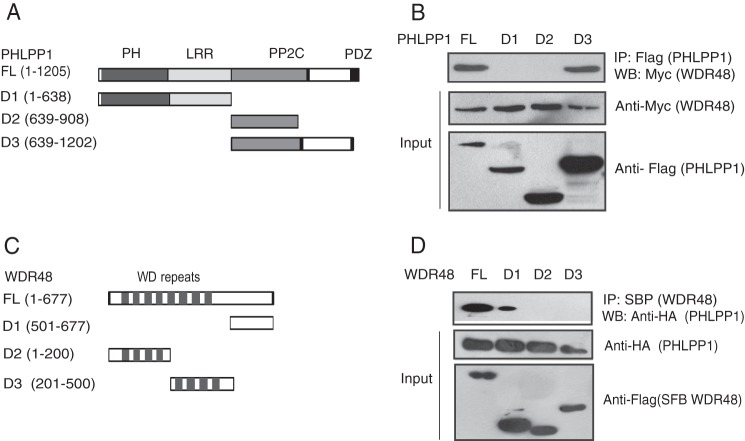
**WDR48 and PHLPP1 interact via their C-terminal regions.** *A*, schematic represents N-terminal SFB-tagged PHLPP1 full-length (*FL*), along with its various deletion mutants. *B*, 293T cells were co-transfected with the SFB-tagged PHLPP1 constructs along with those encoding Myc-tagged WDR48, and the interaction between PHLPP1 and WDR48 was determined by immunoprecipitation (*IP*) and immunoblotting (*IB*) with the indicated antibodies. *C*, schematic represents N-terminal SFB-tagged WDR48 full-length, along with its various deletion mutants. *D*, 293T cells were co-transfected with the SFB-tagged WDR48 constructs along with those encoding HA-tagged PHLPP1, and the interaction between PHLPP1 and WDR48 was determined by immunoprecipitation and immunoblotting with the indicated antibodies.

In addition to WDR48, we also observed that deubiquitinating enzyme USP12 in the list of PHLPP1-associated proteins. As PHLPP1 was already known to be ubiquitinated by β-TrCP (11), we hypothesized that WDR48-USP complex may participate in removing these ubiquitin chains by interacting with PHLPP1. We confirmed the association of endogenous PHLPP1 with WDR48 and USP12 by immunoprecipitation with PHLPP1 antibody (Fig. 1*B*). Interestingly, we observed that PHLPP1 specifically associated with WDR48 and USP12 but not other USPs (Fig. 1*C*). Although USP1 (12) and USP46 (13) were recently shown to deubiquitinate PHLPP1, we did not observe any interaction with these DUBs in our experiments, suggesting that WDR48 might be specifically recruiting USP12 as a major deubiquitinase to regulate PHLPP1 in these cells or their interaction might be very weak, which we could not detect in our experimental conditions. We further confirmed the existence of a specific PHLPP1·WDR48/USP12 complex *in vivo* by demonstrating that WDR48 and USP12 but not other WD repeat protein WDR5 co-immunoprecipitated with exogenously expressed PHLPP1 in 293T cells (Fig. 1*D*). In addition, bacterially expressed GST-PHLPP1 but not GST alone pulled down WDR48 (Fig. 1*E*). PHLPP1 has several domains that are critically important for its function. To identify the WDR48 interacting region of PHLPP1 we generated a series of N-terminal or C-terminal deletion mutants of PHLPP1 that lack different domains (Fig. 2*A*). Co-expression of these constructs along with full-length Myc-tagged WDR48 followed by immunoprecipitation suggests that WDR48 interacts with PHLPP1 within a region between its phosphatase domain and PDZ domain (residues 908–1202 amino acids) (Fig. 2*B*). However, to map the interacting region of PHLPP1 in WDR48 we also generated deletion mutants of WDR48, which contain or lack WD repeats (Fig. 2*C*). Co-expression of these constructs along with full-length PHLPP1 followed by immunoprecipitation suggested that the C-terminal region (devoid of WD repeats) is required for its binding to PHLPP1 (Fig. 2*D*).

##### WDR48·USP12 Complex Regulates PHLPP1 Protein Stability by Deubiquitination

**FIGURE 3.**
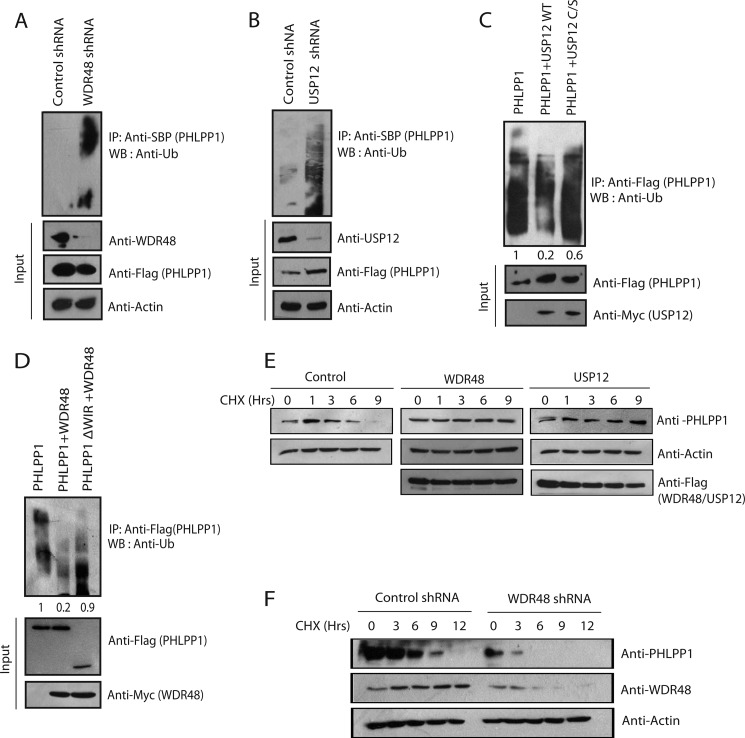
**WDR48·USP12 complex regulates PHLPP1 protein stability by deubiquitination.** *A*, HCT116 cells were transfected with either control shRNA or shRNA against WDR48. Cell lysates prepared after 6 h of MG132 (10 μm) treatment were subjected to immunoprecipitation (*IP*) with streptavidin beads (*SBP*), and the ubiquitinated PHLPP1 was detected with anti-ubiquitin (*Ub*) antibody. The protein expression and the efficiency of shRNA were confirmed by immunoblotting (*WB*) of cell extracts using antibodies as indicated. *B*, HCT116 cells were transfected with control shRNA or shRNA against USP12 shRNA. Ubiquitination of PHLPP1 was detected as described in *A. C*, Myc-tagged wild type USP12 or catalytically inactive C48S mutant of USP12 was expressed in HCT116 cells along with FLAG-PHLPP1. The levels of PHLPP1 ubiquitination were evaluated by immunoprecipitation of PHLPP1 using anti-FLAG antibody followed by immunoblotting with anti-ubiquitin antibody. The values presented below the ubiquitin blot were normalized ubiquitinated PHLPP1/input PHLPP1 derived from the quantification of the blots by using Image Lab software. *D*, Myc-tagged wild type WDR48 was expressed in HCT116 cells along with either full-length FLAG-PHLPP1 or PHLPP1 ΔWIR mutant (WDR48 interaction-deficient mutant). PHLPP1 ubiquitination was evaluated using anti-ubiquitin antibody after immunoprecipitation with FLAG antibody. The values presented below the ubiquitin blot were normalized ubiquitinated PHLPP1/input PHLPP1 derived from the quantification of the blots by using Image Lab software. *E*, HEK293T cells were transfected with plasmids encoding SFB-tagged WDR48 and USP12. Twenty four hours after transfection, cells were treated with cycloheximide (*CHX*; 50 μg/ml) and collected at the indicated times. The protein levels of PHLPP1 were determined by immunoblotting with PHLPP1 antibody. *F*, HCT116 cells were transfected with control and WDR48 shRNA. The levels of PHLPP1 were detected using specific antibody at the indicated times after cycloheximide treatment.

Because WDR48 is known to assemble and activate deubiquitinases to regulate its downstream substrates, we further tested whether the WDR48·USP12 complex controls PHLPP1 ubiquitination. Knockdown of WDR48 by using specific shRNA resulted in significant accumulation of polyubiquitinated PHLPP1 (Fig. 3*A*). Similarly, USP12 knockdown also resulted in increased accumulation of PHLPP1 ubiquitinated species (Fig. 3*B*), suggesting that WDR48-associated USP12 might be required for deubiquitination of PHLPP1. The enzymatic activity of USP12 is required for PHLPP1 deubiquitination as PHLPP1 was significantly deubiquitinated by wild type but not the catalytically inactive USP12 (USP12 C48S) (Fig. 3*C*). However, PHLPP1 deubiquitination was enhanced by overexpression of full-length WDR48 but not the mutant (ΔWIR) that lacks the region of PHLPP1 interaction, indicating that PHLPP1 deubiquitination was dependent on its interaction with WDR48 (Fig. 3*D*).

Because polyubiquitination of PHLPP1 was known to regulate its protein stability, we further assessed whether the WDR48·USP12 complex-mediated deubiquitination of PHLPP1 alters its protein levels. In a cycloheximide chase experiment, we observed that PHLPP1 levels were stabilized to a greater extent in presence of WDR48 and USP12 compared with PHLPP1 alone (Fig. 3*E*). In contrast, shRNA-mediated knockdown of WDR48 drastically reduced the protein half-life of PHLPP1 (Fig. 3*F*), clearly suggesting that the WDR48·USP12 complex stabilizes PHLPP1 by promoting its deubiquitination.

##### WDR48 and USP12 Negatively Regulate Akt Signaling and Promote Apoptosis

**FIGURE 4.**
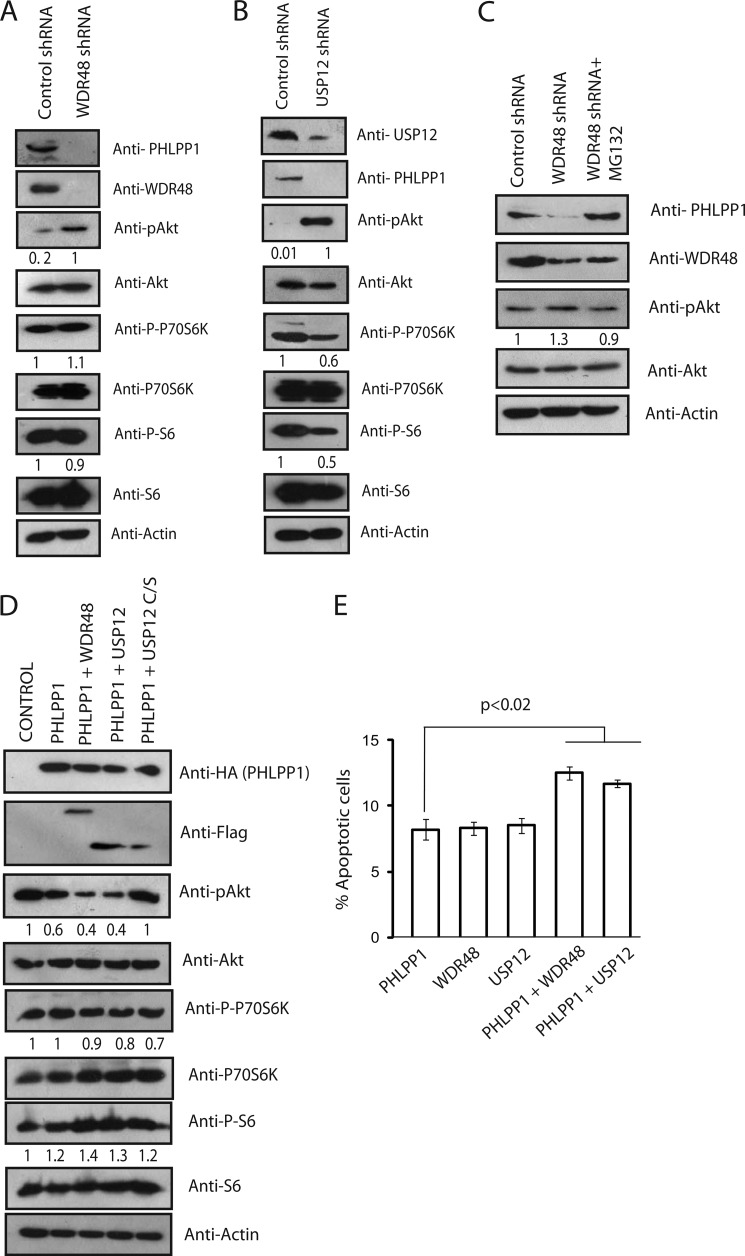
**WDR48 and USP12 negatively regulate Akt signaling.** *A*, HCT116 cells were transfected with either control shRNA or WDR48 shRNA. 48 h after shRNA transfection, cells were lysed, and Akt activation was evaluated by immunoblotting with p-Akt (Ser-473)-specific antibody. Expression of other proteins was detected by immunoblotting with their respective antibodies as indicated. The values presented below representative blots indicate the normalized phosphorylated protein/total protein derived from the quantification of the blots by using Image Lab software. *B*, HCT116 cells were transfected with either control shRNA or USP12 shRNA, and the phospho-Akt, phospho-p70 S6 kinase, phospho-S6 levels along with their total protein levels were detected by using their specific antibodies. The normalized data for each phosphoprotein/total protein are indicated. *C*, HCT116 cells were transfected with control shRNA or WDR48 shRNA alone or in combination with MG132 treatment. Forty-eight hours after shRNA transfection, cells were lysed, and the levels of PHLPP1, p-Akt, and total Akt activation were evaluated by immunoblotting with their respective antibodies. *D*, HCT116 cells were transfected with either PHLPP1 or in combination with wild type WDR48, USP12, or C48S mutant of USP12. The cells were lysed, and the activation of Akt was detected by Western blotting with phospho-specific (Ser-473) Akt antibody. Expression of other proteins was detected by immunoblotting with their respective antibodies as indicated. *E*, HCT116 cells were transfected with PHLPP1, WDR48, and USP12 alone or in the indicated combination. Forty-eight hours transfection the cells were collected, and apoptosis was measured by propidium iodide staining followed by flow cytometric analysis. *Error bars* indicate S.D. (*n* = 3); *p* < 0.02, Student's *t* test.

Because PHLPP1 is a potent negative regulator of the PI3-kinase/Akt pathway, we hypothesized that WDR48 and USP12 might suppress Akt signaling by stabilizing PHLPP1. To test this hypothesis, we depleted WDR48 by using shRNA and analyzed the activation of Akt. Indeed, knockdown of WDR48 led to significant increase in the levels of phosphorylated Akt (Fig. 4*A*). Similarly, shRNA-mediated knockdown of USP12 also resulted in increased activation of Akt (Fig. 4*B*) with no apparent changes in the total Akt levels. Surprisingly, no significant changes were observed in phosphorylation of p70 S6 kinase and S6 ribosomal proteins upon knockdown of WDR48 and USP12, possibly suggesting that the WDR48 complex may mediate its functions mainly through an Akt-dependent but p70 S6K- and S6-independent manner. The destabilization of PHLPP1 and activation of AKT upon depletion of WDR48 are dependent in proteosomal activity as these events could be restored with MG132 treatment (Fig. 4*C*). As knockdown of WDR48 and USP12 increased Akt activation, we next tested whether WDR48 and USP12 could potentiate the suppression of Akt signaling by PHLPP1. In fact, overexpression of WDR48 and USP12 together with PHLPP1 resulted in reduction of Akt phosphorylation, supporting the synergistic effect of the WDR48·USP12 complex along with PHLPP1 in suppressing Akt signaling (Fig. 4*D*). The synergistic effect of the WDR48·USP12 complex with PHLPP1 in suppressing Akt signaling is again dependent on the catalytic activity of the deubiquitinase as inactive mutant of USP12 shows no synergy in reducing Akt activation.

It is well known that PHLPP1 being a tumor suppressor promotes apoptosis by negatively regulating Akt. Because the WDR48·USP12 complex stabilized PHLPP1 and acted in synergy with PHLPP1 in reducing Akt activation we hypothesized that expression of these proteins might also result in cellular apoptosis. In fact, overexpression of WDR48 and USP12 induced apoptosis similarly to PHLPP1. In agreement with their synergy with PHLPP1 function, simultaneous expression of WDR48 and USP12 along with PHLPP1 in cells led to significant increase in apoptosis compared with expression of PHLPP1 alone (Fig. 4*E*). Thus, these results suggest that WDR48 and USP12 negatively regulate Akt signaling and thereby induce apoptosis in conjunction with PHLPP1.

##### WDR48·USP12 Is a Potential Tumor Suppressor Complex

**FIGURE 5.**
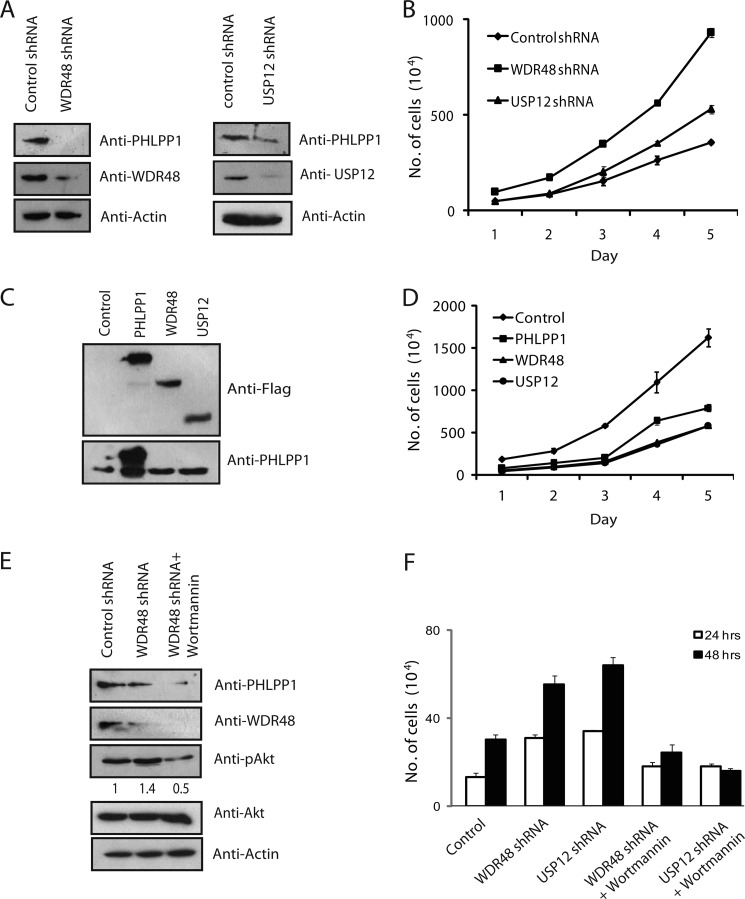
**WDR48 and USP12 negatively affect tumor cell proliferation.** *A*, HCT116 cells were transfected with control shRNA, WDR48 shRNA, or USP12 shRNA, and expression of PHLPP1, WDR48, and USP12 was detected by using their respective antibodies. *B*, cells described in *A* were seeded, and their proliferation was measured by trypan blue exclusion for 5 days. *Error bars* indicate S.D. (*n* = 3); *p* < 0.01 for all shRNA; Student's *t* test was used. *C* and *D*, HCT116 cells expressing PHLPP1, WDR48 or USP12 were seeded (*C*), and cell proliferation was measured by trypan blue exclusion for 5 days (*D*). *Error bars* indicate S.D. (*n* = 3); *p* < 0.01, Student's *t* test. *E*, HCT116 cells expressing control shRNA, WDR48 shRNA alone or along with wortmannin (3 nm) treatment were checked for expression of WDR48, PHLPP1, and Akt activation. *F*, cells described in *E* were seeded, and their proliferation was measured by trypan blue exclusion for 24 and 48 h. *Error bars* indicate S.D. (*n* = 3); *p* < 0.01 for all shRNA, Student's *t* test.

PHLPP1 acts as a tumor suppressor by negatively regulating the Akt pathway. Thus, WDR48·USP12, being a deubiquitinase complex and a positive regulator of PHLPP1 stability, might function as a tumor suppressor complex as well. To test this possibility, we depleted WDR48 and USP12 by shRNA in HCT116 cells (Fig. 5*A*) and analyzed their rate of proliferation. As shown in Fig. 5*B*, knockdown of WDR48 and USP12 by shRNA accelerated the rate of cell proliferation compared with control shRNA-transfected cells. In contrast, overexpression of WDR48 and USP12 (Fig. 5*C*) suppressed cell proliferation similarly to PHLPP1 (Fig. 5*D*).

By using inhibitor studies we further tested whether WDR48-mediated suppression of cell proliferation is dependent on Akt inactivation. In fact, the augmented Akt activation and cell proliferation observed upon knockdown of WDR48 and USP12 were significantly reduced by treatment of cells with wortmannin (Fig. 5, *E* and *F*), suggesting that inactivation of Akt is a crucial downstream event for WDR48-controlled cell proliferation.

##### A Cancer-associated WDR48 (L580F) Mutant Is Defective in Regulating PHLPP1

**FIGURE 6.**
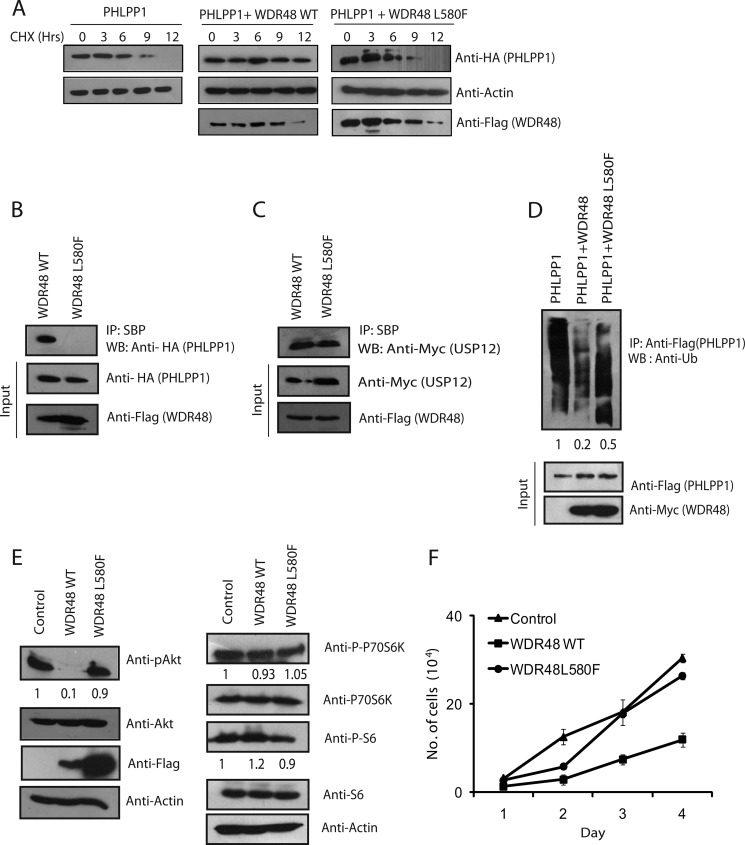
**Colorectal cancer-associated WDR48 (L580F) mutation is defective in stabilizing PHLPP1.** *A*, HCT-116 cells expressing HA-tagged PHLPP1 were transfected with plasmids encoding SFB-tagged WDR48 WT and WDR48 L580F mutant. Twenty-four hours after transfection, cells were treated with cycloheximide (*CHX*; 50 μg/ml), and the protein levels of PHLPP1 at the indicated times were determined by anti-HA immunoblotting. *B*, 293T cells were transfected with wild type WDR48 (*WT*) or L580F mutant along with HA-PHLPP1, and their interaction was evaluated by immunoblotting (*WB*) with anti-HA antibody after immunoprecipitation (*IP*) with streptavidin beads. *C*, 293T cells were transfected with wild type WDR48 or L580F mutant along with Myc-USP12, and their interaction was evaluated by immunoblotting with anti-myc antibody after immunoprecipitation with streptavidin beads. *D*, SFB-tagged PHLPP1 was expressed in HCT116 cells along with either wild type WDR48 or L580F mutant. PHLPP1 ubiquitination was evaluated using anti-ubiquitin antibody after immunoprecipitation with FLAG antibody. The values presented below ubiquitin blot are normalized ubiquitinated PHLPP1/input PHLPP1 derived from the quantification of the blots by using Image Lab software. *E*, HCT116 cells transfected with either wild-type WDR48 or L580F mutant were lysed, and the activation of Akt was detected by Western blotting with phospho-specific (Ser-473) Akt antibody. Expression of other proteins was detected by immunoblotting with their respective antibodies as indicated. The values presented below representative blots indicate the normalized phosphorylated protein/total protein derived from the quantification of the blots by using Image Lab software. *F*, HCT116 cells (10^4^) expressing WDR48 WT or L580F mutant were seeded, and cell proliferation was measured by trypan blue exclusion for 4 days. *Error bars* indicate S.D. (*n* = 3); *p* < 0.01, Student's *t* test.

To further establish the role of WDR48 as a tumor suppressor and its dependence on PHLPP1 regulation, we screened for WDR48 mutations in human cancers that might be defective in regulating this pathway. Our search for somatic mutations in the COSMIC database revealed several missense and nonsense mutations in WDR48. Among these mutations, we identified that an L580F mutation of WDR48 found in colon adenocarcinoma is defective in stabilizing PHLPP1 (Fig. 6*A*). Because the mutation is located in the C-terminal PHLPP1 interacting region of WDR48, we hypothesized that mutant inability to stabilize PHLPP1 might be due to the loss of their interaction. In fact, the WDR48 L580F mutant is severely defective in binding with the substrate PHLPP1 compared with wild type WDR48 (Fig. 6*B*). However, the mutant interaction with USP12 is unaffected (Fig. 6*C*). Consistent with the interaction data, unlike wild type WDR48 the L580F mutant is unable to effectively deubiquitinate PHLPP1 (Fig. 6*D*). In addition, L580F mutant is defective in suppressing Akt phosphorylation unlike wild type WDR48 (Fig. 6*E*). Further, the L580F mutant significantly failed to suppress the rate of tumor cell proliferation (Fig. 6*F*) compared with wild type WDR48. Together, these results suggest that WDR48 acts as a potential tumor suppressor by stabilizing PHLPP1, and mutations that disrupt its interaction with substrates may contribute to tumorigenesis.

## DISCUSSION

In summary, we identified WDR48 and USP12 as two novel players in PHLPP1 regulation. We demonstrated that the WDR48·USP12 complex deubiquitinates and regulates PHLPP1 levels, which is essential for their possible tumor suppressor function. Because PHLPP1 is an important player in controlling cell survival and metabolism, it is critical to maintain the homeostasis of its protein levels and function in the cell. Any alterations in its levels and/or function may lead to diseases. For instance, loss or reduced PHLPP1 promotes proliferative pathways and is frequently associated with cancer, whereas gain of PHLPP1 results in termination of metabolic signaling pathways and neuronal cell survival pathways, which are often associated with insulin resistance in diabetes (22) and neurological disorders, respectively (23).

So far, very limited mechanisms have been identified for regulation of PHLPP1 in cells. Whereas regulation of PHLPP1 at transcript level is completely unknown, its regulation at translational and post-translational levels is reported. At the translational level PHLPP1 is controlled by an mTOR-dependent pathway whereas at the post-translational level it is regulated by calpain-mediated proteolytic cleavage and β-TrCP-mediated proteasomal degradation. However, the enzymatic machinery is required to maintain proper PHLPP1 protein levels in cells by opposing β-TrCP mediated PHLPP1 degradation. USP1 (12) and USP46 (13) were recently identified as deubiquitinases for PHLPP1. Nevertheless, because PHLPP1 functions in various tissues derived from different cellular origins these regulators may not fully elucidate the molecular players controlling PHLPP1 function. This might be in agreement with the fact that multiple ubiquitin/deubiquitin pathways may co-exist in different cellular contexts to maintain optimum levels of a crucial molecular players in normal cells and pathological conditions. For example, important tumor suppressors such as p53 and PTEN have been shown to be controlled by multiple enzymes in the ubiquitin pathway in a cellular context-dependent manner (24, 25).

Although WDR48 has been well established as an activatory subunit of different deubiquitinase complexes such as USP1, USP12, and USP46, its complete cellular functions and role in human malignancies have not been clearly demonstrated. In our study, in addition to identifying WDR48 as a physical partner of PHLPP1 in cells, functionally we also demonstrated its ability to act as a potential tumor suppressor. In fact, we identified a disease-associated WDR48 mutation in colon adenocarcinoma that is defective in binding and maintaining optimal PHLPP1 levels in cells. Other studies have previously shown that WDR48 is found in complex with different proteins such as PCNA, BRCA1, p53, FANCD2, and FANCI (17, 26), all of which play a very critical role in maintenance of genome stability and tumor suppression. Thus, WDR48 might function as tumor suppressor by positively regulating the stability of several tumor suppressor proteins in the cell. In the future it would be very interesting to screen different human cancers for additional WDR48 mutations that might not only be defective in maintaining PHLPP1 function but also other substrates such as BRCA1, FANCD2, H2A/H2B, PCNA, and notch receptor.

## Footnotes

3 The abbreviations used are:

PHLPP1: PH domain leucine-rich repeat protein phosphatase 1
PCNA: proliferating cell nuclear antigen
SFB: S-protein/FLAG/streptavidin-binding protein.
